# The complete mitogenome of the *Lepturacanthus savala* (Perciformes: Trichiuridae) from the Yellow Sea

**DOI:** 10.1080/23802359.2020.1789512

**Published:** 2020-07-15

**Authors:** Chuangeng Cai, Na Song, Linlin Zhao, Tianxiang Gao

**Affiliations:** a Fisheries College, Ocean University of China, Qingdao, China; b First Institute of Oceanography, Ministry of Natural Resources, Qingdao, China; c College of Fisheries, Zhejiang Ocean University, Zhoushan, China

**Keywords:** Mitochondrial genome, *Lepturacanthus savala*

## Abstract

*Lepturacanthus savala* is a commercially important marine fish belonging to the family Trichiuridae, with a wide distribution in the Indian-Western Pacific coastal waters. In this study, the complete mitochondrial genome of *L. savala* has been determined by primer walking methods. The mitogenome is 17,146 bp in length containing 13 protein-coding genes, 2 ribosomal RNA genes, 21 transfer RNA genes, a control region, and an origin of light-chain replication region. The base composition is 28.9% for A, 29.0% for C, 15.7% for G, and 26.4% for T. The maximum-likelihood tree based on whole mitogenome showed that it is the closest related species of *Trichiurus.*

The genus *Lepturacanthus* Fowler, 1905 belongs to the family Trichiuridae and comprises three valid species: *Lepturacanthus savala* (Cuvier, 1829), *Lepturacanthus pantului* (Gupta, 1966), and *Lepturacanthus roelandti* (Bleeker, 1860), features very elongated and strongly compressed body with a ribbon-like caudal fin, gradually tapering to a point (Nakamura and Parin 1993; Chakraborty et al. 2006). In this study, we present the complete mitochondrial genome of *L. savala*, which has been deposited in GenBank with accession no. MT269921.

Samples were collected from Qingdao, Yellow Sea (120°46.064′, 36°22.452′) in 10 November 2017. Specimens were preserved in 95% ethanol and deposited at the Fish Specimen Room of Fisheries Ecology Laboratory (specimen accession no. OUC_FEL201910051), Fisheries College, Ocean University of China, Qingdao City, Shandong Province, China. Total genomic DNA was isolated from the muscle tissue by proteinase K digestion followed by the standard phenol–chloroform method (Sambrook and Russell 2001). PCR primer sets were designed using Primer Premier 6.0 (PREMIER Biosoft International, Palo Alto, CA) based on the complete mitochondrial genome sequences of *Trichiurus nanhaiensis* (GenBank accession no. NC018791) and *Trichiurus japonicus* (GenBank accession no. MK292708). The normal PCR was performed following the standard procedure (Liu et al. 2006). The tRNA genes were predicted using online software tRNAScan-SE (Lowe and Chan 2016).

The complete mitogenome of *L. savala* is 17,146 bp in length, containing 13 protein-coding genes, 21 tRNA genes, 2 rRNA genes, a control region (D-Loop), and an origin of light strand replication (O_L_). Different with typical 22 tRNA genes in vertebrate mitogenome, the mitogenome contained 21 tRNA genes except for tRNA^Pro^. The tRNA^Pro^ gene is absent also in *T. japonicus* and *T. nanhaiensis* (Liu and Cui 2009; Liu et al. 2013; Xu et al. 2019; Zheng et al. 2019). Most of the genes are transcribed on the heavy strand (H-strand), except for seven tRNA genes (Gln, Ala, Asn, Cys, Tyr, Ser-UGA, Glu). The base composition is 28.9% for A, 29.0% for C, 15.7% for G, and 26.4% for T, with a slight AT bias of 55.3%. The small non-coding region, a light-chain replication region (OL) was observed between the tRNA^Asn^ and tRNA^Cys^ in WANCY region and it could form a stable stem-loop secondary structure. The largest non-coding region (control region) located between the tRNA^Thr^ and tRNA^Phe^ genes is determined to be 1485 bp in length. Within the control region, termination associated sequence (TAS), central conserved domain (CSB-D, E, F), conserved sequence block (CSB-1, 2, 3), as well as two different types of tandem repeat sequences were detected respectively.

Maximum-likelihood phylogeny of *L. savala* and other 17 Scombriformes fishes based on complete mitochondrial genomes were reconstructed, using *Bactrocera dorsalis* (DQ845759) as an outgroup in the MEGA 7 software (Kumar et al. 2016). This phylogenetic tree shows that *L. savala* is the closest related species of *Trichiurus* with high bootstrap value supported (Figure 1).

**Figure 1.**
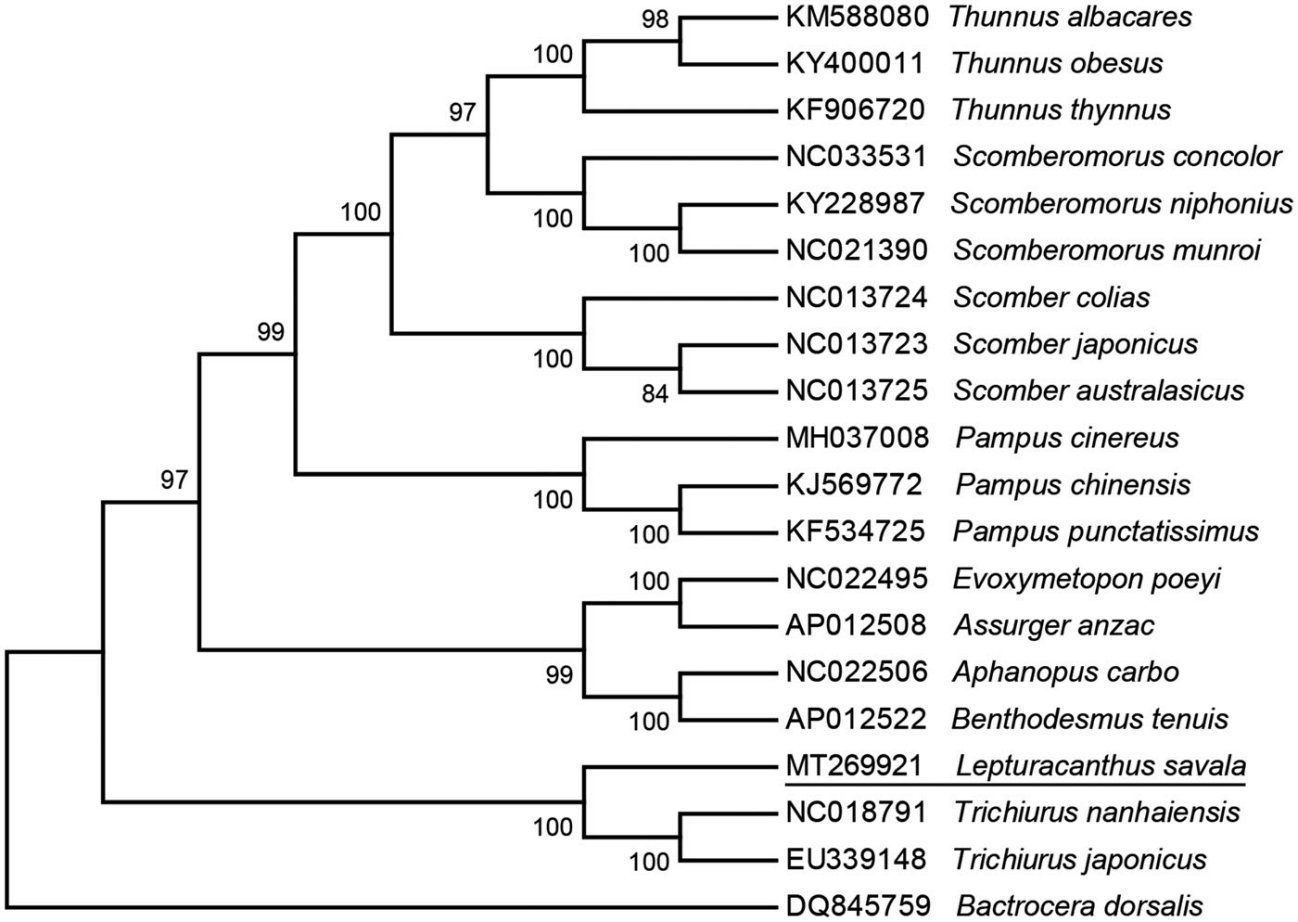
Phylogenic tree of *Lepturacanthus savala* and the related species based on complete mitogenome by maximum-likelihood method with 1000 bootstrap replicates. The position of *L. savala* is underlined.

## Data Availability

The data that support the findings of this study are openly available in GenBank at https://www.ncbi.nlm.nih.gov/genbank/, accession number [MT269921].
